# Australia

**Published:** 1896-07

**Authors:** 


*Australia.* Queensland has adopted measures to limit the ravages of Texas fever by dipping the cattle in an iron solution for the destruction of the ticks. Another method, claimed to be very effective in destroying these parasites, is the use of a mixture applied with brushes and brooms..

